# Interventions to Promote Early Discharge and Avoid Inappropriate Hospital (Re)Admission: A Systematic Review

**DOI:** 10.3390/ijerph16142457

**Published:** 2019-07-10

**Authors:** Alice Coffey, Patricia Leahy-Warren, Eileen Savage, Josephine Hegarty, Nicola Cornally, Mary Rose Day, Laura Sahm, Kieran O’Connor, Jane O’Doherty, Aaron Liew, Duygu Sezgin, Rónán O’Caoimh

**Affiliations:** 1 Department of Nursing and Midwifery, Health Sciences Building, University of Limerick, Limerick V94X5K6, Ireland; 2 School of Nursing and Midwifery, University College Cork, Cork City T12AK54, Ireland; 3 Nursing and Vice Dean of Graduate Studies and Inter Professional Learning, College of Medicine and Health, University College Cork, Cork City T12AK54, Ireland; 4 School of Pharmacy, University College Cork, Cork City T12T656, Ireland; 5 Geriatric Medicine, Mercy University Hospital, Cork City T12WE28, Ireland; 6 Clinical Sciences Institute, National University of Ireland, and Portiuncula University Hospital, Ballinasloe Galway H53T971, Ireland; 7 Clinical Sciences Institute, National University of Ireland, Galway City, Mercy University Hospital, Grenville Place, Cork City T12WE28, Ireland

**Keywords:** discharge, admission, primary care, length of stay, transition, intermediate care, homecare, model, intervention, hospital avoidance

## Abstract

Increasing pressure on limited healthcare resources has necessitated the development of measures promoting early discharge and avoiding inappropriate hospital (re)admission. This systematic review examines the evidence for interventions in acute hospitals including (i) hospital-patient discharge to home, community services or other settings, (ii) hospital discharge to another care setting, and (iii) reduction or prevention of inappropriate hospital (re)admissions. Academic electronic databases were searched from 2005 to 2018. In total, ninety-four eligible papers were included. Interventions were categorized into: (1) pre-discharge exclusively delivered in the acute care hospital, (2) pre- and post-discharge delivered by acute care hospital, (3) post-discharge delivered at home and (4) delivered only in a post-acute facility. Mixed results were found regarding the effectiveness of many types of interventions. Interventions exclusively delivered in the acute hospital pre-discharge and those involving education were most common but their effectiveness was limited in avoiding (re)admission. Successful pre- and post-discharge interventions focused on multidisciplinary approaches. Post-discharge interventions exclusively delivered at home reduced hospital stay and contributed to patient satisfaction. Existing systematic reviews on tele-health and long-term care interventions suggest insufficient evidence for admission avoidance. The most effective interventions to avoid inappropriate re-admission to hospital and promote early discharge included integrated systems between hospital and the community care, multidisciplinary service provision, individualization of services, discharge planning initiated in hospital and specialist follow-up.

## 1. Introduction

Safe and timely discharge and the avoidance of (re)admission are important markers of the quality of acute hospital care, and signs of effective integration between hospital and community services [1]. Economic and logistical pressures on acute hospital resources in public healthcare systems are a phenomenon seen across the developed world. The potential negative consequences of this on patient outcomes and experiences during care transitions have been identified [2]. Delayed discharge occurs when a patient remains in hospital after they have been documented as fit to leave [2]. This may be due to various reasons including poor decision-making and information sharing structures, or the lack of suitable discharge destinations outside the hospital [3]. Furthermore, when a patient is clinically ready for discharge, but cannot leave hospital, the reasons may be due to a lack of access to care, support or accommodation outside the hospital [4]. Inappropriate (re)admission is defined as an unplanned return to hospital after being discharged within a set interval often defined as the last thirty days [5]. Inappropriate (re)admission is in some instances avoidable, particularly where it results from problems such as poor discharge processes from a previous hospital stay or poor care at the discharge destination [4,6,7]. Delayed discharge and avoidable (re)admission increase exposure to iatrogenic harm, slows the flow of patients through the hospital system, and are a strain on hospital resources [1]. A wide range of interventions have attempted to address these problems, reflecting the complex and multifactorial nature of their origin [8,9,10,11]. 

In the last two decades, there has been an influx of published literature on the different aspects of transitioning older persons from acute services to home. Some of the research includes patients being ready for discharge [12,13,14,15], interventions to improve the process [16,17,18] and review of how acute and community services intersect [14,19,20]. Various models of service delivery have been illustrated, including care related to rehabilitation and intermediate care within the United Kingdom [21] and hospital at home in the United States [22]. Although there have been many previous systematic reviews of individual interventions, there is no single review that brings all the evidence together. In this paper, we critically review existing interventions designed to promote early discharge and avoid inappropriate hospital (re)admission.

## 2. Methods

### 2.1. Design

The review methods used in this paper were guided by methodology used in systematic review as outlined by the University of York [23]. The Preferred Reporting Items for Systematic Review and Meta-Analyses (PRISMA) statement was used as a reporting guide [24]. The main outcome of this systematic review was to identify interventions that are developed and implemented to prevent delayed discharge and avoid inappropriate hospital (re)admissions. Therefore, the following research questions were investigated:

(1) What type of interventions were developed and implemented to promote early discharge, prevent and manage delayed discharge, and/or avoid inappropriate hospital (re)admission?
(2) What are the components of the identified interventions?

### 2.2. Eligibility Criteria 

Articles included in this review were selected using the Population, Intervention, Comparison Outcome and Studies (PICOS) framework. Inclusion criteria are presented in detail in Supplementary Materials S1. Patients from all ages were included so that the scope of interventions used for early discharge could be reviewed. Peer reviewed publications were reviewed and scrutinized and were only selected if they met the following inclusion criteria: specific studies which focused on interventions related to acute hospital setting (hospitals focused on treating patients with a brief episode of illness) which included: (i) in-patient discharge from acute hospital to home, community service or any other setting, (ii) discharge from one in-patient setting to another and (iii) reduction or prevention of inappropriate hospital admissions. Peer reviewed empirical papers which focused on interventions related to (re)admission avoidance were included if measurements of discharge or rates of (re)admission were explicitly stated as primary outcomes.

### 2.3. Information Sources

A search strategy was developed using a number of keywords: bed utilizations; length of stay; intermediate care; homecare; residential care; primary care; model; intervention; technology; hospital avoidance; delayed; transition; admission and discharge. Subject headings relevant to the individual databases were also applied as needed. The following databases were searched: PsychINFO, SocIndex, MEDLINE, Social Sciences, CINAHL, Psychology and Behavioural Sciences Collection. Details of the full electronic search strategy and their strings are described in Supplementary Materials S1. Searches were limited to papers in the English language and published during the period of January 2005 and 2018. Papers were limited to these constraints in order to reflect recent changes and developments within the area. The updated search was limited to papers only published in the English language and published between 2015 and 2018. Covidence was used to facilitate the sharing of papers and the screening process [25].

### 2.4. Study Selection Strategy

**Figure 1:**
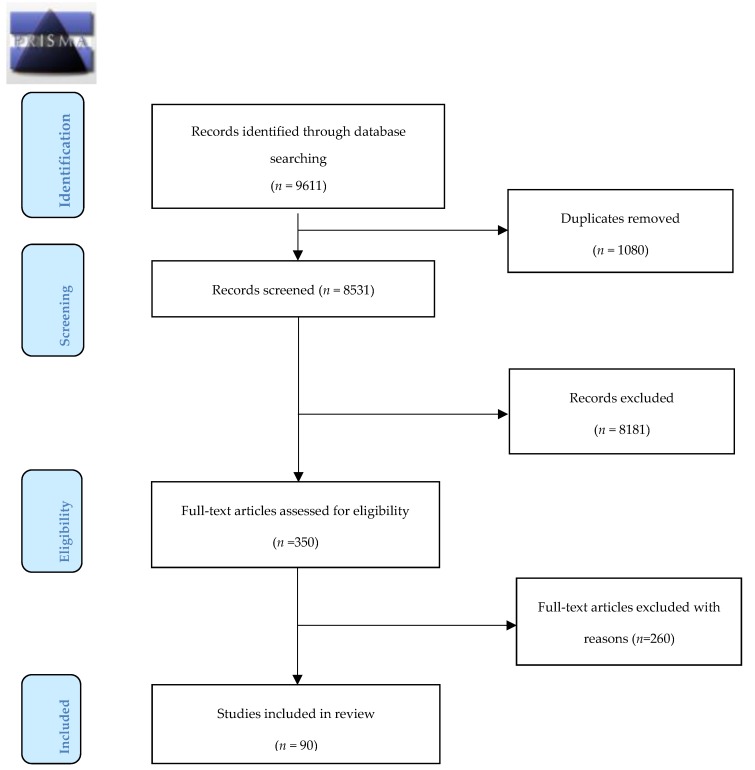
Preferred Reporting Items for Systematic Review and Meta-Analyses (PRISMA) diagram.

For the initial search, findings were reviewed independently by teams of two reviewers who selected papers according to eligibility criteria. When disagreement occurred, a third reviewer was introduced to reach consensus. Firstly, papers were reviewed by title and abstract. Secondly, potentially eligible papers were reviewed (Figure 1 and Supplementary Materials S2: Characteristics of Included Studies.).

### 2.5. Data Extraction, Quality Assessment and Synthesis

Data were extracted from the selected papers based on the review questions: authors and date; type of paper; aims of the paper; delayed discharge definitions; definitions of admission, transition and (re)admission; components of models/interventions/policy; sample size and population of interest; healthcare setting/context; healthcare personnel; outcomes reviewed; effects of outcomes; characteristics associated with positive outcomes within the intervention; resource issues; enablers and barriers to discharge from hospital and (re)admission avoidance. Quality of studies and recommendations by authors were also reviewed and noted. A condensed table showing the data extracted from the included papers is presented in Supplementary Materials S2. Studies were categorized according to the type of intervention studied and are described in detail below. 

Each study was appraised using the Crowe Critical Appraisal Tool (CCAT). Scoring categories include the following categories: preliminaries, introduction, design, sampling, data collection, ethical matters, results and discussion [25,26]. CCAT facilitates the appraisal of a diversity of research designs using the same evaluative tool. All categories are scored, regardless of research design used. The lowest score for a category is 0 (no evidence), the highest score is 5 (highest evidence) and the total score (out of 40 or as a percent) is reported in addition to each category score. CCAT has been extensively validated across studies [25,26].

## 3. Results

In total, 9611 papers were identified of which 1080 were duplicates. The title and abstracts of 8531 papers were screened, which resulted in the exclusion of 8181 papers. Full text review of 350 papers was completed for the remaining papers and this resulted in a further exclusion of 260 papers. Following the review and data extraction, 90 papers met the inclusion criteria. Details of this process are presented in a PRISMA flow diagram (Figure 1). These consisted of one meta-review of meta-analyses, eight systematic reviews with meta-analyses, 33 systematic reviews without meta-analyses, 44 individual RCTs, two retrospective cohort studies, one non-randomized observational study and one intervention study; which focused on pre- and post-intervention results only. Included studies were double-checked for not being covered by the included systematic reviews and meta-analyses. The types of evidence by study design from studies included in this review are categorized and presented in Table 1. The quality assessment of the included studies using the CCAT for assessing risk of bias [25,26] showed that overall, the methodological quality of studies included in this review are good, ranging from 23/40 to 37/40. 

Four settings for the interventions reviewed were classified: interventions exclusively delivered in the acute hospital pre-discharge (*n* = 22), interventions delivered both pre- and post-discharge from acute care (*n* = 23), interventions delivered at home post-discharge from acute care (*n* = 16) and interventions only delivered in a post-acute facility (*n* = 29).

### 3.1. Interventions Exclusively Delivered in the Acute Hospital Pre-Discharge 

Twenty-two papers described interventions which were only delivered in the acute hospital before a patient was discharged. These interventions were quite diverse, and components included patient educational interventions (some of which involved telephone follow-ups), multi-disciplinary clinical management programmes, rehabilitation interventions, and pharmacological care. Patient educational interventions were found to be beneficial in the targeted groups. Pre-discharge interventions occur in the hospital setting and aim to provide a safe and timely transfer to an appropriate destination. Interventions included early discharge planning, nurse-led discharge, nurse-led pre-discharge self-management education, nurse-led post-operative discharge process that are protocol driven, instructional discharge letter from a physician, and discharge medication planning. The main components of these interventions were assessment and education of patients, individualized discharge planning, follow-up telephone calls or visits and a clinical care pathway. Pre-discharge interventions were not found to have a decisive influence on most outcomes. However, one meta-analysis reported that early discharge planning was associated with fewer readmissions, and that length of stay for readmissions was significantly reduced [27]. One study found that point-of-care assessment led to a higher proportion of successful discharges in four hospitals [28], while another study found that hospital costs had an impact on the overall increase in successful discharges [29]. Otherwise, pre-discharge planning was not found to affect readmission rates [30,31,32,33,34] although it was associated with slight improvements in quality of life [27,32] and increased patient understanding of their treatment [32,35]. A systematic review by Ogilvie (2005) found that having acute medical assessment units for children within the hospital, enabled children to be admitted less and had lower rates of readmission [36]. Specific pre-discharge interventions are detailed below.

#### 3.1.1. Education

Hospital readmission rates were reduced following provision of medication and dietary advice, with telephone follow-ups [37]. However, one study which used an educational and screening intervention, for patients with chronic obstructive pulmonary disease (COPD), showed no improvement in readmission rates [38]. It was associated with lower emergency readmission rates among patients with four or more chronic conditions and patients who were on specific medications, i.e., diuretics [39]. In summary, patient educational interventions were the most common clinical intervention. A barrier to successful cardiac rehabilitation included patients’ hesitance to participate in educational and rehabilitation programmes [9,40].

#### 3.1.2. Multidisciplinary Clinical Management Programmes

Multi-disciplinary cardiac rehabilitation was linked to reduced readmission rates, a reduced mortality rate at 12 months post-intervention, and improved quality of life scores, three months post-baseline [40]. However, no improvement in readmission rates was found following the implementation of an intervention comprising of pulmonary rehabilitation and physiotherapy [41]. Similarly, a physiotherapy intervention delivered in an ED extended care unit was found to have no effect on measured outcomes [42]. 

#### 3.1.3. Rehabilitation

One study compared a Community In-reach Rehabilitation and Care Transition (CIRACT) service with traditional hospital-based rehabilitation (THB-Rehab). They found that there was no significant difference in length of stay between the CIRACT and THB-Rehab service [43]. One study involving patients rehabilitating after road trauma was included and indicated that there was no difference in acute length of stay between the intervention and control groups [44].

#### 3.1.4. Pharmaceutical Care

One study involving medication/therapeutics was included, and this demonstrated that for patients with Crohn’s disease, treatment with azathioprine significantly reduced re-hospitalizations, compared with mesalazine treatment [45]. A reduction in admission rates and length of stay were reported following an intervention which utilized radiology, utilizing cardiac stress magnetic resonance imaging (MRI), in patients with suspected acute coronary syndrome [7]. Mixed and non-significant results were found in relation to same day discharge for patients undergoing percutaneous coronary intervention procedures [46]. Length of stay was not influenced whether children received inhalational versus intravenous anaesthesia [46]. A review of RCTs found that children who had received inhalational anesthesia took longer to recover and longer to be discharged from hospital [47].

### 3.2. Interventions Delivered Both Pre- and Post-Discharge from Acute Care

In this review, discharge interventions refer to interventions which begin before hospital discharge and the remainder of the intervention was delivered post-discharge. The interventions discussed here commenced prior to discharge in the acute setting and continued into the home. 

#### Multidisciplinary Clinical Management

Components of interventions included pharmacist-led interventions [48], nurse-driven interventions [49], case management [50], post-discharge telephone calls [51], heart-failure-specific follow-up, and early supported discharge for stroke patients [52]. RCTs also examined the use of a virtual ward model to promote inter-professional community care, nurse-led telephone support, nurse-based case management, and a discharge nurse led intervention to promote self-management of care at home. The core components of these interventions involved patient education, individualized care planning, home visits, telephone contact, management of cases, discharge planning and promotion of self-managed care. One study used clinical pharmacists visiting patients within their homes to reduce readmission rates but the study did not find any difference in the rates of readmission among patients who had pharmacists visit them and home and patients who did not [48].

One study [53] reported on an intervention called “vertical integration”, merging health care providers of different levels into a single unit to reduce fragmentation of care and its negative consequences, especially for older people or those with multiple chronic conditions. After the intervention was completed, the results suggest that readmissions decreased. A systematic review of 19 studies [54] reported a significant decrease in readmissions with studies, and an RCT examining nurse-led discharge planning and telephone support [55] demonstrated a significant difference in readmissions. Similarly, a systematic review and meta-analysis reported that a pharmacist-led/general practitioner (GP) intervention within the hospital setting significantly reduced unplanned admissions for older people with cardiac failure, although it did not improve unplanned readmission rates [56]. Early supported discharge was linked to reduced length of stay within the stroke unit and reduction in the cost of care [57]. Yet another review found significant decreases in admissions to the emergency department (ED) and length of stay [58]. A disease management programme for COPD patients was reported to be linked to a large reduction in hospitalizations and visits to the ED [59]. 

Post-discharge planning interventions appear to affirm a small number of positive results in reducing readmission and unplanned admissions. However, this evidence came only from individual trials, and is not supported by the broader findings of the systematic reviews and meta-analyses. Significant reductions in readmissions were reported in relation to a multidisciplinary community-based care approach, in comparison with standard care [60,61,62,63,64,65,66]. No significant effect was found on ED visits or readmissions using telephone follow-up [66] but increased patient engagement with office visits was noted. Overall, the elements of successful primary care interventions were a multidisciplinary approach, and community-based interventions. In two studies, no improvement was seen in discharges or readmission rates but increased patient engagement was noted [67,68].

### 3.3. Interventions Only Delivered at Home Post-Discharge from Acute Care

Hospital-at-home interventions involve specialist services provided in the individual’s home, and are usually specific to a particular condition. Services included care for adults with COPD [69,70] and a home nursing service for children [71]. Elements of these interventions included frequent home visitations, delivery of treatments at home, symptom and education review. There were conflicting results in relation to readmission rates for adults with COPD [69,70]. Although no difference in readmission rates were found for children receiving hospital at home services, length of hospital stay was decreased and family satisfaction with care was greater [71].

#### Multidisciplinary Clinical Management

Home-based interventions covered more generic aspects of care than the hospital at home interventions and included components such as carer education and support and health promotion. These resulted in fewer hospitalizations and decreased length of stay, among patients following total hip replacement [72]. Participants in the intervention groups were found to be significantly more satisfied with care at home and less likely to be admitted to hospital than those in control groups [73,74]. However, evidence from RCTs demonstrated no differences in length of hospital stay, readmission, use of other health care services, patient mortality or healthcare costs [73,74,75,76,77,78]. A sole home-based educational visit by a nurse, one week after discharge was associated with a reduction in ED visits and unplanned readmissions, improved quality of life, and reduced healthcare costs [74,79,80]. An RCT for patients with cardiovascular disease included a discharge nursing intervention (DNI) but despite this, there was no significant impact on patient’s visiting to hospital [81]. Overall, home-based interventions appeared to offer some positive effects.

Tele-health interventions included remote self-monitoring of vital signs [82,83,84]. Some also included home visits by healthcare professionals [84], remote integrated environmental sensors [83] and electronic discharge systems involving acute and primary care [85,86]. There were no compelling differences found on rates of readmission between the intervention and control groups [82,83,84,85,86]. However, one systematic review reported a decrease in (re)admission in most of the studies they reviewed, and decreased length of stay up to 50% [84].

### 3.4. Interventions in Post-Acute Facility

#### 3.4.1. Ambulatory Care

Assessment or ambulatory care interventions implement a method of accessing out-patient hospital care including diagnostic, treatment and rehabilitation services without need for admission, often following GP referral. Core components of assessment or ambulatory care interventions included individualized assessment by a nurse or geriatrician, quick assessment and diagnosis, review of medication, rehabilitation, case management, education of patients, clinical/tele-monitoring and follow-ups via and advanced care planning. Such interventions generally had a positive effect on most outcomes, with several studies showing significant reduction in (re)admissions following intervention [21,56,68,87,88,89]. However, a ‘Day Hospital’ intervention was shown to double the risk of (re)admission, compared with home rehabilitation [90] and a systematic review found no evidence that comprehensive assessment, benefits older adults discharged from ED or medical assessment units [91]. A cluster RCT by Connolly et al. found that that it is not possible to reduce hospitalizations from residential aged care facilities [92] and in a systematic review by Guerin et al. they found that within four models of discharging patients. These models included Virtual Interface Model (*n* = 6); In-reach Interface Model (*n* = 2); Out-reach Interface Model (*n* = 2); Independent Interface Model (*n* = 2). There was no impact on readmission rates [93]. Fox et al. demonstrated that following these interventions, that patients’ length of stay was shorter and functional decline was less likely [91]. Hospitalizations were also slightly increased among individuals who received specialist geriatric assessment before discharge. One systematic review by Malika et al. and an RCT by Edmans et al. found that geriatric-focused nursing assessment and interventions did not have a statistical impact on hospitalization, readmissions, length of hospital stay and ED revisits [94,95].

#### 3.4.2. Transitional Care

Transitional care interventions focused on managing the transfer from hospital to a post-hospital destination, including home with the aim of preventing avoidable readmission. Interventions included pre- and post-discharge assessments, self-management education, counselling, care coordination, home visits, telephone follow-up, protocols, medication review and co-ordination specialists. They also included referral to services and community supports [96,97]. Transitional care interventions were generally positive in facilitating quicker discharges and reducing readmissions [6,98,99]. However, meta-analyses of RCTs found that despite transitional care being somewhat effective in reducing readmissions overall, this did not occur in the short term [100,101,102,103,104]. Furthermore, a systematic review [96] reported that although readmissions were reduced in COPD patients following transitional care interventions, mortality also increased in the intervention group. Transitional care that included medication management and reconciliation reduced hospital length of stay and readmissions [105]. An RCT conducted by Herfjord et al. found that although transitional care did not reduce the number of patients residing at home significantly, it did reduce need for nursing home and homecare services [106]. A systematic review conducted by Tabanejad et al. found that overall, four of the six studies had a liaison nursing service had significantly positive effects on outcomes measured. Outcomes measured included timing of discharge, readmission rates to the intensive care unit, length of stay within the hospital and patient self-care abilities [107]. Faxed discharge medication care plans did not reduce post-discharge utilization of healthcare services or adverse patient outcomes. Similarly, a nurse-led discharge intervention for cardiac patients had no effect on outcomes [57,58].

#### 3.4.3. Long-Term Care Interventions

Long-term care interventions including outreach residential aged care integration programmes which have shown that rates of acute hospitalization increased for patients in both intervention and control groups [108]. However, one study noted that residents were less likely to require outpatient visits, and had higher satisfaction with care [109]. There were few studies in this category and thus conclusions are tentative.

#### 3.4.4. Pharmaceutical Care

One RCT aimed to determine if a pharmacist case manager (PCM) providing a faxed discharge medication care plan from a tertiary care institution to primary care could improve medication appropriateness and reduce adverse events. There were no statistically significant effects between healthcare groups and healthcare utilization [110].

## 4. Discussion

The objective of this review was to identify all potential interventions that promoted early discharge and avoided inappropriate readmission to acute hospitals. The review included 90 papers spanning classes of intervention before and after hospital discharge. Those exclusively delivered pre-discharge in the acute hospital that included patient-focused educational and rehabilitation programmes may be of some benefit. Early discharge planning also appears to reduce readmission rates. Hospital discharge planning by advanced nurse practitioners influenced reductions in readmissions over longer-term periods, especially when combined with home visits. Similarly, post-discharge planning interventions reduced readmissions in some settings and populations, though results were mixed. Community-based interventions were associated with lower rates of readmissions. Specialized multidisciplinary community-based interventions in primary care showed positive outcomes for patients. Ambulatory care interventions usually focusing on assessment were associated with positive outcomes when they were patient centered and focused on early rehabilitation, early discharge planning and interdisciplinary teamwork. Overall, positive effects were found following home-based interventions or hospital-at-home interventions for older people with chronic disease. There was limited evidence for tele-health and other electronic system approaches in studies of patients recently discharged from acute hospitals. 

There was considerable variation in the extent to which the review’s main questions were answered in relation to the interventions, aims and healthcare professionals involved. Very few studies addressed the issue of resources and their barrier and enablers. Also, this review sought models/interventions which tackled two related concepts; delayed discharge and (readmission) avoidance. Barriers to successful intervention were numerous and broad and thus difficult to identify, however, those common across care contexts included: costs; insufficient time for patient education; difficulties agreeing upon the most appropriate professional to lead; lack of acute assessment skills and variation in intensity of home visiting. Many of the studies included in this review had a focus on specific health issue such as COPD and a specific cohort of patients. Accordingly, the results cannot be applied to the general population of patients who are faced with being readmitted to hospital, for example older people. Studies were clinically and methodologically heterogenous and there were few common outcome measures available, for example, rate of readmission to hospital and unplanned admission were defined and measured differently, making comparison between studies challenging. Despite this, it is possible based on the as yet limited results, to suggest that groups of interventions with complementary components that are more likely to be successful in promoting early discharge and in avoiding hospital re-admission. 

### Strengths and Limitations 

This systematic review conformed to international best practice guidelines as proposed by the PRISMA group. Papers that were included provided a high level of evidence, which was assessed by two independent reviewers using the CCAT. A multidisciplinary team from the disciplines of nursing, medicine and pharmacy conducted this review. It provides a comprehensive report addressing the aim and objective, and addresses a considerable number of other questions. Therefore, to our knowledge, this systematic review is unique in both outlook and focus. There are also limitations. As with any systematic review, the search was not exhaustive and is prone to publication bias. Further, a number of studies may have been missed because the search was confined to publications in English. Data were heterogeneous, meaning that a meta-analysis was not possible to conduct within the scope of this review. Meta-analysis was not performed due to the inherent clinical and methodological heterogeneity of the included studies. Long-term follow-up data are needed to determine if interventions will have a sustained impact on patients being discharged earlier from hospital. The increased growth and change in this area, both clinically and academically, over recent years will no doubt provide a platform for future intervention studies.

## 5. Conclusions

Overall, the evidence for the effectiveness of interventions to promote early discharge and avoid inappropriate hospital (re)admission in acute hospitals is mixed. Despite this, our systematic review found positive effects from key components within these interventions. Key elements for which there was evidence and that merit further study include integrated systems spanning acute and home-based services; multidisciplinary care provision; person-centered services; and discharge initiated in acute care pre-discharge initiated with specialist follow-up. This review suggests that these areas should receive particular focus in future research, and also highlights a need for a consensus on definitions and agreed structures to support this. Trials that do not use clear definitions are likely to be unhelpful and difficult to compare and reproduce. Specific lessons for clinicians and policy-makers are that positive effects on (re)admission avoidance can result from early discharge planning in hospital, patient-focused education in hospital which continues at home, post-discharge support continuing from hospital including telephone follow-up; integrating the hospital and community care, and transitional care structures with access to a multifaceted multidisciplinary team.

## Figures and Tables

**Table 1:** Types of evidence.

Type of Study	Interventions Exclusively Delivered in the Acute Hospital Pre-Discharge	Interventions Delivered Pre- and Post-Discharge from Acute Care	Interventions only Delivered at Home (Post-Discharge from Acute Care)	Interventions only Delivered in a Post-Acute Facility
MR				1
MA				
SR & MA	1	2		4
SR	1	9	7	14
RCT	18	7	9	10
Non-randomised trial		2		
Pre–post-intervention study		1		
Retrospective cohort		2		

MR = Meta-review; MA = Meta-analysis; SR = Systematic review; RCT = Randomized controlled trial.

## Supplementary Materials

The following are available online at https://www.mdpi.com/1660-4601/16/14/2457/s1, Supplementary Materials S1: Final Search Strings; Supplementary Materials S2: Characteristics of Included Studies.
